# System Integrated Digital Empowering and teleRehabilitation to promote patient Activation and well-Being in chronic disabilities: A usability and acceptability study

**DOI:** 10.3389/fpubh.2023.1154481

**Published:** 2023-03-28

**Authors:** Federica Rossetto, Francesca Borgnis, Sara Isernia, Emanuela Foglia, Elisabetta Garagiola, Olivia Realdon, Francesca Baglio

**Affiliations:** ^1^IRCCS Fondazione Don Carlo Gnocchi ONLUS, Milan, Italy; ^2^School of Industrial Engineering and Healthcare Datascience LAB, LIUC-Università Carlo Cattaneo, Castellanza, VA, Italy; ^3^Department of Human Sciences for Education, Università degli Studi di Milano-Bicocca, Milan, Italy

**Keywords:** eHealth, telerehabilitation, usability, acceptability, chronic disability, Parkinson's disease, chronic obstructive pulmonary disease, chronic heart failure

## Abstract

**Introduction:**

Telerehabilitation systems represent a promising way for the management of chronic disability, delivering technology-enabled rehabilitation outside the hospital setting. However, usability and acceptability assessment with users represents a critical starting point when using digital healthcare solutions. This study aims at evaluating the user experience with a Telerehabilitation system (SIDERA^∧^B) from the end-user side.

**Methods:**

SIDERA^∧^B consists of an asynchronous delivery of rehabilitation activities through multimedia digital contents and tele-monitoring of vital parameters with technological devices for individualized, home-based management of chronic conditions. Usability (with the System Usability Scale, SUS) and acceptability (using the Technology Acceptance Model, TAM - and The Service User Technology Acceptance Questionnaire, SUTAQ) data were analyzed from the dataset of the SIDERA^∧^B project (*N* = 112 patients with Chronic Heart Failure, Parkinson's Disease and Chronic Obstructive Pulmonary Disease). The possible influence of five external factors (i.e., technological expertise, education, sex, age, and level of disability) on TAM domains was tested using Spearman's Correlation analysis.

**Results:**

Results showed a satisfactory level of technological usability (SUS Median = 77.5) and good scores in usability and learnability SUS subdomains (mean scores > 2.5). Regarding technological acceptability, participants showed high scores (Median > 4) in “Behavioral Intention”, “Perceived Usefulness”, and “Perceived Ease of Use” TAM domains. Finally, results from the SUTAQ scale highlighted that the SIDERA^∧^B system obtained optimal scores in all domains, especially in “Increased accessibility,” “Care personnel concerns,” and “Satisfaction.” Age (rho = −0.291, *p* = 0.002) and disability level (WHODAS Total score: rho = −0.218, *p* = 0.021) were the two external factors inversely associated with the Perceived Ease of Use.

**Discussion:**

The age of digital transformation requires everyone to understand, accept and master the changes affecting modern-day healthcare. The usability and acceptability of the SIDERA^∧^B system were high across all end-users, despite the medium-low level of the technological expertise of the sample. These findings support the efficiency and the suitability of these digital solutions in the modern digital age transition of rehabilitation from inside to outside the clinic.

## Introduction

Telerehabilitation has recently led to numerous advantages in the field of health, improving the efficiency of medical practice and guaranteeing easier and continued access to healthcare services (1, 2). It consists in the provision of technology-enabled rehabilitation interventions outside the hospital setting through a “double-loop” communication between the clinic and the patient's home (3–7). However, for an effective use, technologies in healthcare require rigorous validation to prove their usability and acceptability in addition to clinical benefits (8). These factors may have a great impact on the user's inclination to use telerehabilitation systems.

According to Brooke “*we could define the usability of a particular artifact as the appropriateness to a purpose of that specific artifact”* (9). As a consequence, the usability of a system must be assessed considering the context in which it will be used and the end-users of that system. In fact, the usability assessment offers insight into “*the degree to which a subject is able to use a system to achieve specific goals effectively, efficiently, and within a well-defined context of use*” (10). Notably, a recent scoping review (2019) included the lack of technology usability and technical support as critical barriers to digital health adoption (11). Technological systems with poor usability can lead to situations of low goal-achievement efficiency or the technology not being used or being rejected (12). Specifically, technology abandonment may occur when users decide that telerehabilitation technology is too difficult to learn or requires high maintenance levels (13).

Usability is not the only factor having an impact on the system use. A number of variables may determine people's acceptance or rejection of digital solutions. The “acceptability” of technology can be considered a higher-level concept compared to usability and serves as a tradeoff among all those factors affecting the adoption of new technologies (14). According to the Technology Acceptance Model [TAM (15, 16)], two key dimensions may determine if a technology is more likely to be accepted by users: the “*perceived ease of use*”, representing the degree to which a person believes that the use of a technological tool will be effortless, and the “*perceived usefulness*” indicating the belief that the technological tool is capable of being used advantageously or help to perform better an activity. These two main beliefs may influence the “*behavioral intention*”, which is the user's inclination to use the technology. Recently, Tsertsidis and colleagues (2019) have detailed additional external factors influencing the user acceptance of technology, including demographic characteristics, benefits experienced with technology (e.g., increased safety, health condition, independency, capabilities to perform everyday activities), technological expertise (subjects with more experience with technology are more likely to adopt innovative technologies), and social/cultural influences (17). Moreover, Hirani and colleagues (2017) identified a number of variables specifically referable to the acceptability of telehealth solutions, including usability, accessibility, comfort, privacy and security, confidentiality, satisfaction, convenience, health benefits, and self-care (18). Overall, acceptability models agree that several external factors (e.g., age, education, technological expertise, or disability level) could limit the use of digital health solutions (1, 19, 20). Therefore, the user experience with technology should be assessed by testing both usability and acceptability (13) as a critical starting point in developing and using telerehabilitation solutions (1, 21).

This study aims to evaluate the user experience with a telerehabilitation program supported by innovative technologies for patients with chronic disabilities including Chronic Heart Failure (CHF), Chronic Obstructive Pulmonary Disease (COPD), and Parkinson's Disease (PD), the “*System Integrated Digital Empowerment and Rehabilitation to promote patient Activation and well-Being*” [SIDERA^∧^B; (22)]. This telerehabilitation system consists of an asynchronous delivery of rehabilitation activities through multimedia digital contents and tele-monitoring of vital parameters with technological devices for individualized, home-based management of chronic conditions. The usability and acceptability of the telerehabilitation system will be investigated from the end-user side considering the technological solutions adopted, such as apps, sensors and wearable devices integrated into the medical platform for the self-monitoring and self-management of health conditions. Moreover, the user experience with health technologies will be evaluated considering the effect of external variables, such as demographic characteristics, technological expertise, and the level of disability linked to both neuromotor and cardio-pulmonary diseases.

## Materials and methods

In this study, we tested the perceived usability and acceptability of a telerehabilitation system from data collected within the SIDERA^∧^B project (Lombardy Region, POR-FESR 2014–2020, I.1.B.1.3, https://www.liuc.it/ricerca/ricerca-accademica/progetti/siderab-sistema-integrato-domiciliare-e-riabilitazione-assistita-al-benessere/).

### Participants and telerehabilitation system

Subjects included in the analysis (*n* = 112) were selected from the entire SIDERA^∧^B dataset (*N* = 141) filtered according to the following criteria: (1) people with a diagnosis of Chronic Heart Failure (CHF) according to European Society of Cardiology guidelines (23), or of Chronic Obstructive Pulmonary Disease (COPD) according to the American Thoracic Society (ATS) and the European Respiratory Society (ERS) criteria (24), or of Parkinson's Disease (PD) according to the Movement Disorder Society (MDS) criteria (25); (2) without a cognitive impairment condition [Montreal Cognitive Assessment test - MoCA test < 17.54 (26)]; (3) who fully attended the telerehabilitation program (lasting 3 month for CHF and 4 months for COPD and PD) between September 2019 to September 2020; (4) who completed all the usability and acceptability scales and questionnaires during the in-clinic evaluation session; and (5) who read and signed the written informed consent approved by the “IRCCS Fondazione Don Carlo Gnocchi-Milan” Ethics Committee.

The telerehabilitation system consisted of: (1) the SIDERA^∧^B digital platform (clinical side); (2) the home-based kit (patient side). The digital platform combines a telerehabilitation module with telemonitoring of vital parameters and health status and a tele-engagement module for wellbeing. The home-based technological kit consists of a tablet with the SIDERA-app for delivering individualized daily rehabilitation activities and medical devices for vital signs monitoring (i.e., activity tracker, blood pressure monitor, balance, and pulse oximeter). Each telerehabilitation session involved three multidimensional activities: Endurance Training, Resistance Training, and Neuromotor Training [for more detail, see (22)]. To improve the quality of care at home, these training modules were digitalized (app) with the aim to foster internal adaptive loops for self-management in an asynchronous modality and to guarantee monitoring from the clinical staff.

### Materials

Data included in the dataset were inherent to participants' characteristics, perceived usability, and technology acceptability.

#### Participants characteristics

Data on subjects' characteristics included in the analysis were demographics (i.e., age, education, and sex), the level of disability before treatment measured with the WHO Disability Assessment Schedule 2.0 [WHODAS 2.0: World Health Organization, 2004; (27)], and the individual technological expertise evaluated with an *ad-hoc* questionnaire exploring the frequency of use of technological devices (i.e., Personal Computer and Tablet) in daily life. The items of this *ad-hoc* questionnaire range between 1 (“everyday”) and 7 (“never”); therefore, a low value reflects a high frequency of use of technology.

#### Perceived usability

The usability assessment was performed using the System Usability Scale [SUS, (9, 28–30)], a valid, reliable, and quick-to-use scale widely implemented to evaluate the usability of an extensive range of technological devices. SUS is a short questionnaire on a 5-point scale from “completely disagree” to “strongly agree”. The SUS score ranges from 0 to 100 and indicates the system's overall usability (9, 30, 31). The obtained scores were evaluated according to the scale's score acceptability ranges (cut off = 68) and mapped in six adjective rating scales according to Bangor et al. (28): “worst imaginable” (0–25); “poor” (25.1–51.6); “OK” (51.7–71); “good” (71.1–80.7), “excellent” (80.8–84), and “best imaginable” (84.1–100). Finally, SUS allows for evaluation of the two main aspects that can affect the user experience: *usability*, which indicates the ease with which the user uses the system (scores 1–4), and *learnability*, which represents the ease with which the user learns to use the system (scores 1–4) [51].

#### Acceptability assessment

The acceptability assessment was conducted through the Technology Acceptance Model 3 questionnaire [TAM3, (32)] and the Service User Technology Acceptance Questionnaire [SUTAQ, (18)].

TAM3 is a 7-points Likert scale questionnaire (ranging from 1- strongly disagree to 7-strongly agree) able to identify the “behavioral intention” underlying the true use of the technology. Specifically, we focused the attention on two main beliefs that influence the user's inclination to use technology: (1) “Perceived ease of use”: the degree to which a person believes that the use of a technological tool will be effortless; and (2) “Perceived usefulness”, the belief that, by using the tool, the user will improve their productivity.

The SUTAQ is a 6-point Likert scale questionnaire (ranging from 1-strong disagreement and 6-strong agreement) used to evaluate the perception of the acceptability of technological treatments in telemedicine (18). In more detail, the questionnaire is composed of six different domains in which low scores reflect a negative perception of telemedicine, concerning specific aspects of the service:

1. “Enhanced care”: patients' concerns about their health status, their perception of active involvement, recommendations to people in a similar condition and the perception of improved care;
2. “Increased accessibility”: patients' perception of saving time, greater access to care, improved health and easier contact with professionals;
3. “Privacy and discomfort”: patients' concerns about privacy and their perception of discomfort;
4. “Care personnel concerns”: perception by patients of the continuity of care and concerns relating to the staff involved in the service;
5. “Satisfaction”: patient satisfaction and understanding of telemedicine services;
6. “Kit as substitution”: patients' concerns about their state of health and their perception of the service as a substitute for regular care and face-to-face consultations.

The items composing the “Privacy and discomfort” and “Care personnel concerns” domains have been reversed to align the range score with the remaining items; therefore, for all domains, a low value reflects a negative perception.

### Statistical analysis

All statistical analyses were performed using Jamovi 2.2.5 software [The jamovi project (2021). *jamovi*. (Version 2.2) https://www.jamovi.org.]. A statistical threshold of *p* < 0.05 was considered statistically significant. The normality of data distribution was assessed using the Shapiro-Wilk test, and non-parametrical analyses were performed accordingly.

#### Participants characteristics

Descriptive statistics including frequencies, percentages median, and interquartile range (IQR) were reported to describe the participants' characteristics. Also, Kruskal-Wallis and Chi-square tests were conducted to verify possible differences between groups in demographic data (age, education, and sex), level of disability and technological expertise.

#### Usability assessment

Descriptive statistics were used to investigate the usability level in the three chronic conditions and the Kruskal-Wallis test was run to verify possible differences between groups in usability scores.

#### Acceptability assessment

Descriptive statistics and the Kruskal-Wallis test were performed to report acceptability variables level and possible differences between groups. The possible influence of five external factors (technological expertise, education, sex, age, and level of disability) on “Perceived Usefulness” and “Perceived Ease of Use” TAM domains that impact the “Behavioral Intention” was observed using Spearman's Correlation.

## Results

### Participants characteristics

Table 1 reports the main characteristics of the included subjects (*N* = 112), divided into the three clinical conditions (PD, COPD, CHF). The three groups are comparable for all main demographic characteristics, respectively, sex, education, and age and for the level of disability. Regarding the technological expertise of participants, our results showed a medium-low level of technological expertise in all clinical groups.

**Table 1:** Sample characteristics.

	**PD**	**COPD**	**CHF**	**TOT**	**Groups comparison (*p*-value)**
*N* (%)	45 (40.18%)	34 (30.36%)	33 (29.46%)	112 (100%)	0.305
Age (years—Median; IQR)	70.9; 14.30	73.1; 9.98	68.1; 14.10	70.6; 5	0.413
Education (years—Median; IQR)	13; 5	10.5; 5	8; 5	10.5; 5	0.427
Sex (M:F)	24:21	24:10	24:9	72:40	0.138
WHODAS total score (Median; IQR—range 0–100)	16.46; 17.91	18.37; 19.95	12.64; 17.92	15.95; 18.67	0.347
Technological expertise (Median; IQR—range 1–7)	4; 5.25	4.25; 3	5; 3	4.5; 4	0.187

PD, Parkinson's disease; COPD, chronic obstructive pulmonary disease; CHF, chronic heart failure; TOT, total sample (PD + COPD + CHF). *N*, number; IQR, interquartile range; M, males; F, females; WHODAS, WHO Disability Assessment Schedule 2.0.

### Usability assessment

Table 2 reports the usability assessment scores of the cohort (*N* = 112), divided into the three clinical conditions (PD, COPD, CHF). Data show good technological usability for all clinical populations. Statistical differences appear between COPD and PD in the Total SUS score [$\chi_{\left(2\right)}^{2}$ = 6.85, *p* = 0.033] and in the usability dimension [$\chi_{\left(2\right)}^{2}$ = 6.26, *p* = 0.044].

**Table 2:** Usability scores of the sample.

	**PD**	**COPD**	**CHF**	**TOT**	**Groups comparison (*p*-value)**	***Post-hoc***
Total SUS score (median; IQR)	72.5; 22.50	82.5; 26.30	75; 12.50	77.5; 22.50	**0.033**	PD < COPD
Adjective rating scale score (median; IQR)	4; 2	5; 3	4; 1	4; 3	0.076	–
Usability_score (median; IQR)	2.88; 0.87	3.31; 0.75	2.88; 0.62	3; 0.78	**0.044**	PD < COPD
Learnability_score (median; IQR)	3; 1.50	4; 1.50	3; 1	3, 2	0.075	–

PD, Parkinson's disease; COPD, chronic obstructive pulmonary disease; CHF, chronic heart failure. TOT, total sample (PD + COPD + CHF); IQR, interquartile range. In bold, values statistically significant (Kruskal-Wallis).

Considering the adjective rating scale, our results indicate that the majority of patients (85.7%) rate the SIDERA^∧^B system as usable (adjective rating score ≥ 3). Table 3 reports the percentage-based scores for each adjective rating of the SUS, considering the three clinical conditions.

**Table 3:** Percentage-based scores for each adjective rating of the SUS, considering the three clinical conditions.

	**Worst imaginable**	**Poor**	**OK**	**Good**	**Excellent**	**Best imaginable**
PD (*N*; %)	1; 2.2%	5; 11.1%	16; 35.6%	11; 24.4%	2; 4.4%	10; 22.2%
COPD (*N*; %)	2; 5.9%	3; 8.8%	5; 14.7%	4; 11.8%	5; 14.7%	15; 44.1%
CHF (*N*; %)	–	5; 15.2%	9; 27.3%	11; 33.3%	2; 6.1%	6; 18.2%
TOT (*N*; %)	3; 2.7%	13; 11.6%	30; 26.8%	26; 23.2%	9; 8.0%	31; 27.7%

PD, Parkinson's disease; COPD, chronic obstructive pulmonary disease; CHF, chronic heart failure; TOT, total sample (PD + COPD + CHF); *N*, number.

### Acceptability assessment

Table 4 shows the scores of SUTAQ domains about the telerehabilitation treatment comparing the three clinical conditions (PD, COPD, CHF). Statistical differences appear between groups in the Enhanced Care [$\chi_{\left(2\right)}^{2}$ = 13.35, *p* = 0.001; COPD < PD and CHF], in Privacy and discomfort [$\chi_{\left(2\right)}^{2}$ = 15.92, *p* < 0.001; CHF > COPD and PD], in Care Personnel Concern [$\chi_{\left(2\right)}^{2}$ = 8.21, *p* = 0.017; COPD > PD and CHF], in Satisfaction [$\chi_{\left(2\right)}^{2}$ = 8.37, *p* = 0.015; PD < COPD] and in Kit as substitution [$\chi_{\left(2\right)}^{2}$ = 6.20, *p* = 0.045; CHF > COPD].

**Table 4:** Comparison between three clinical conditions in SUTAQ domains.

**SUTAQ domains**	**PD**	**COPD**	**CHF**	**TOT**	**Group comparison**	***Post-hoc***
Enhanced care (median; IQR)	4.50; 0.75	4; 0.94	4.75; 0.5	4.5; 1	**0.001**	PD > COPD; COPD < CHF
Increased accessibility (median; IQR)	4.75; 1	5.13; 0.69	4.75; 1.5	5; 1	0.071	–
Privacy and discomfort (median; IQR)	5.33; 1	5.5; 1.83	6; 1	5.33; 1.34	**<0.001**	PD < CHF; COPD < CHF
Care personnel concerns (median; IQR)	5.67; 0.33	6; 0.33	5.67; 0	5.67; 0.67	**0.017**	PD < COPD; COPD > CHF
Satisfaction (median; IQR)	5.33; 1	5.67; 0.67	5.33; 1	5.33; 1.33	**0.015**	PD < COPD
Kit as substitution (median; IQR)	4; 2	3; 3	4; 1	4; 2	**0.045**	COPD < CHF

PD, Parkinson's disease; COPD, chronic obstructive pulmonary disease; CHF, chronic heart failure; TOT, total sample (PD + COPD + CHF); IQR, interquartile range. In bold, values statistically significant (Kruskal-Wallis).

The analyses conducted on TAM3 showed that patients (*N* = 112) attributed high average scores (>4 - neutral value) to all domains considered in the analyses. Specifically, considering the two main beliefs that influence the user's inclination to use the technology, 81.2% of patients attributed high scores to the “Perceived Usefulness” domain, and 78.6% awarded high scores to the “Perceived Ease of Use” domain.

Table 5 shows the scores obtained on the TAM3 domains considering the three clinical conditions (PD, COPD, CHF). Statistical differences appear between groups in the Perceived Ease of Use [$\chi_{\left(2\right)}^{2}$ = 6.14, *p* = 0.046]. However, the pairwise *post-hoc* comparison did not reach statistical significance.

**Table 5:** Comparison between the three clinical conditions in the TAM3 domains.

**TAM domains**	**PD**	**COPD**	**CHF**	**TOT**	**Group comparison**
Behavioral intention (median; IQR)	5; 0.7	5; 0.7	4.67; 0.67	5; 0.67	0.712
Perceived usefulness (median; IQR)	6; 1.5	6.50; 1.06	6; 1.25	6.25; 1.44	0.068
Perceived ease of use (median; IQR)	6; 1.25	6.38; 1.31	5.50; 1.88	6; 1.5	0.046*

PD, Parkinson's disease; COPD, chronic obstructive pulmonary disease; CHF, chronic heart failure; TOT, total sample (PD + COPD + CHF); IQR, interquartile range. In bold, values statistically significant (Kruskal-Wallis). ^*^Pairwise *post-hoc* comparison did not reach statistical significance.

### The role of external factors on perceived usefulness, perceived ease of use and behavioral intention

**Figure 1:**
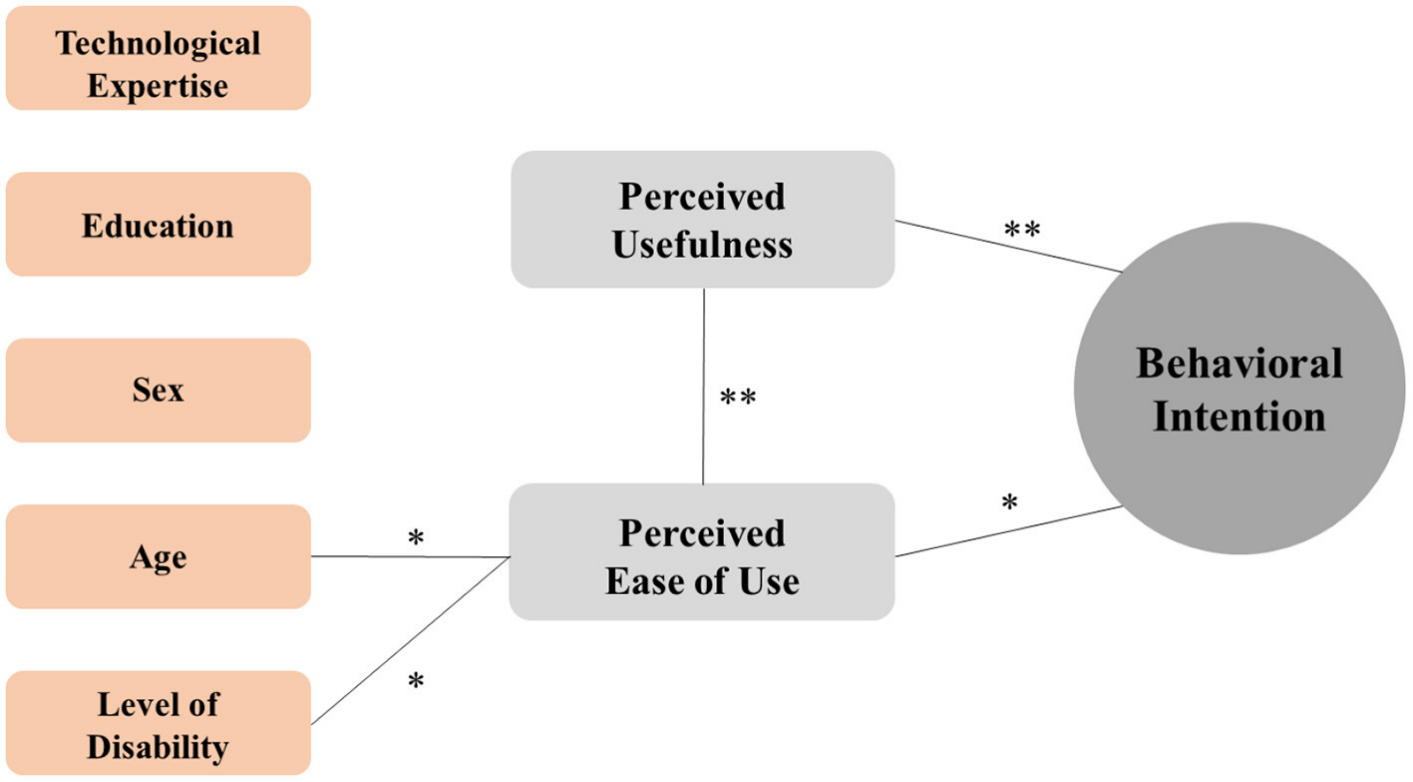
Diagrammatic representation of the relationship between factors influencing Behavioral Intention in the whole sample (PD, COPD, CHF). Only the statistically significant correlation and related magnitude were reported by connection lines between TAM components (gray boxes) and/or external factors (orange boxes). **p < 0.001; *p < 0.05.

Figure 1 shows factors influencing Behavioral Intention in the whole sample (PD, COPD, CHF). A significant correlation emerged between Behavioral Intention and the two main beliefs influencing the user's inclination to use technology, “Perceived Usefulness (rho = 0.340, *p* < 0.001) and Perceived Ease of Use (rho = 0.272, *p* = 0.004). A significant correlation between these two beliefs (rho = 0.675, *p* < 0.001) was shown. Moreover, age (rho = −0.291, *p* = 0.002) and disability level (WHODAS Total score: rho = −0.218, *p* = 0.021) were the external factors inversely associated with the Perceived Ease of Use. Technological expertise, education and sex did not impact both TAM beliefs.

**Figure 2:**
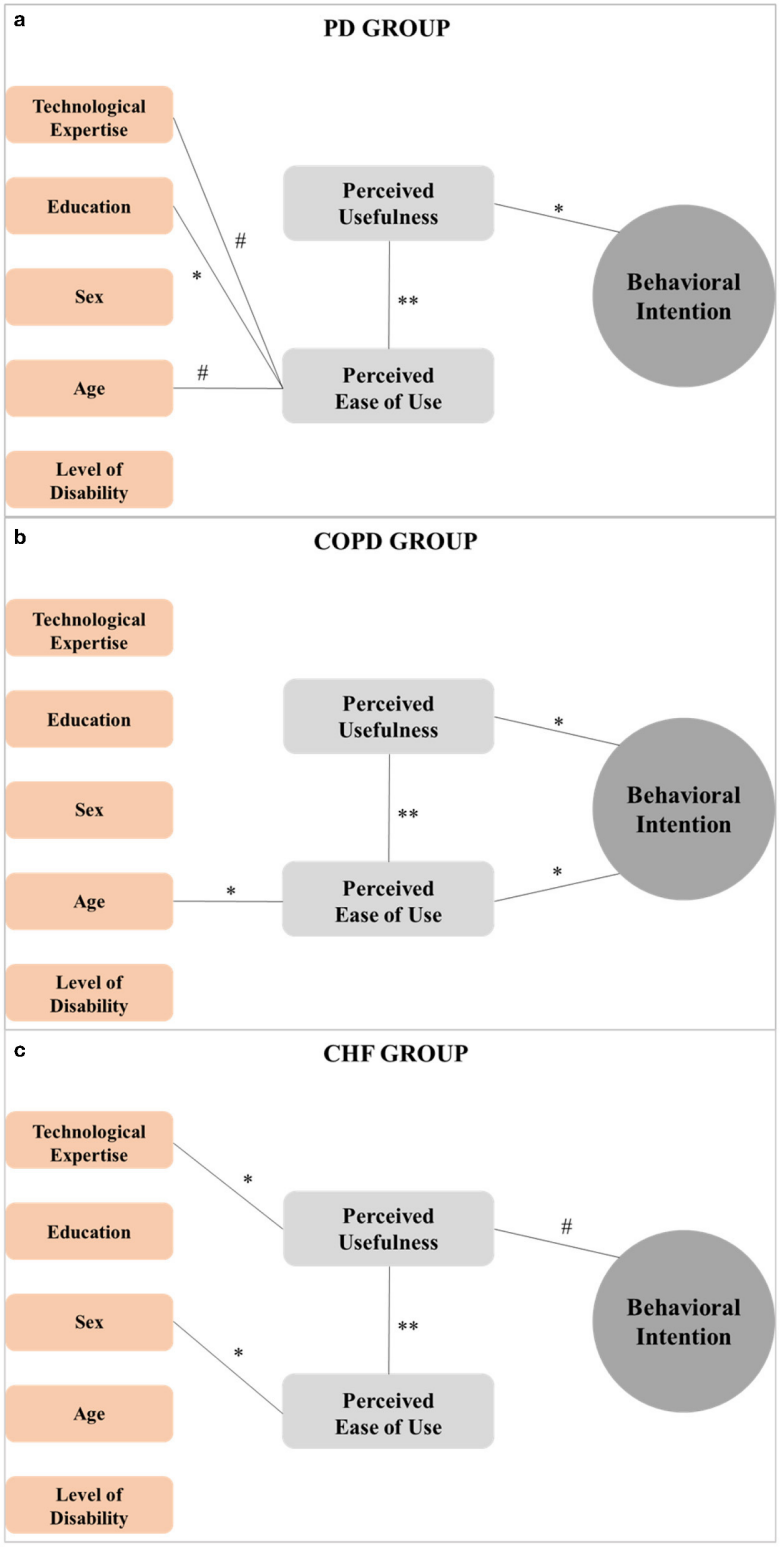
Diagrammatic representation of the relationship between factors influencing Behavioral Intention in the PD **(a)**, COPD **(b)**, and CHF **(c)** groups. Only the statistically significant correlation and related magnitude were reported by connection lines between TAM components (gray boxes) and/or external factors (orange boxes). **p < 0.001; *p < 0.05; ^#^ <0.06.

Considering differences between the clinical conditions, Figure 2 shows factors influencing Behavior Intention respectively in the PD (Panel a), COPD (Panel b) and CHF (Panel c) groups. The external factors affecting Behavioral Intentions differed among the three diseases. Considering demographic characteristics, age was the only significant external factor inversely associated with the Perceived Ease of Use in COPD (rho = −0.436, *p* = 0.010), and showed a trend with Perceived Ease of Use in PD (rho = −0.293, *p* = 0.051). Sex significantly affected Perceived Ease of Use in CHF (rho = 0.384, *p* = 0.027). Education significantly correlated with Perceived Ease of Use only in PD (rho = 0.299, *p* = 0.046).

Technological expertise was a significant external factor (rho = −0.370, *p* = 0.034) associated with Perceived Usefulness in CHF while showing a trend of association with the Perceived Ease of Use in PD (rho = −0.292, *p* = 0.052)

Data showed that Perceived Usefulness significantly correlates with Behavioral Intention in PD (rho = 0.303, *p* = 0.043) and COPD (rho = 0.486, *p* = 0.004) groups, showing a trend of association in CHF. Only in the COPD group, a significant correlation appeared between Perceived Ease of Use and Behavioral Intention (rho = 0.456, *p* = 0.007). Finally, data showed a significant association between Perceived Usefulness and Perceived Ease of Use in all clinical conditions (*p* < 0.001).

## Discussion

The present study aimed at investigating the user experience of people with different types of chronic disabilities in the interaction with a telerehabilitation system named SIDERA^∧^B. This system provides for motor telerehabilitation activities and telemonitoring of vital parameters in the asynchronous modality, in which the patient and the therapist do not interact in real-time (3), assuring considerable advantages for patients (6, 7). It has been recently demonstrated that Telerehabilitation, especially in the asynchronous modality, positively impacts clinical outcomes in chronic clinical conditions, increasing and maintaining functional capacity and quality of life, and promoting adherence to treatment (6, 7). Subjects included in this study were ideal candidates for asynchronous telerehabilitation, considering that they were both adults and seniors with different types of disability at their initial phase, in absence of relevant cognitive deterioration and with mild levels of physical impairment. Moreover, their technological expertise was medium-low, allowing us to evaluate the user experience with an asynchronous telerehabilitation system, in people quite unfamiliar with the technologies. It is well-known that digital skills are weaker among seniors, as they often do not have an eHealth device.

Regarding the usability of the telerehabilitation system, we observed high levels of perceived technological usability, with most participants (85.7%) rating the telerehabilitation system as a suitable solution. This result is notable since the literature reported that the efficacy of digital health solutions is strictly related to the perceived ease of use of health care systems (33). In detail, the good perceived usability level of the telerehabilitation system proposed is associated with a high learnability experience: patients evaluated the system as “easy-to-learn” as much as “easy-to-use”. Specifically, patients claimed that only a few elements need to be learned to make the best use of the system and that “*most people would learn to use the SIDERA*^∧^*B system very quickly*”. Globally, the high level of perceived usability of the telerehabilitation system is relevant considering our participants' characteristics, presenting old age (median age of 70.6), medium-low educational level and low technological expertise (use of technology approximately once a month). In particular, several studies highlighted that age, education, and previous familiarity and competence with technologies potentially affect the user's experience with digital health solutions (1, 19, 20). For this reason, considering such demographic factors is mandatory along all design and implementation phases of telerehabilitation systems. Indeed, usability, referred to as appropriateness of a specific artifact to a purpose (9, 30), requires that user interaction with a technological device should be designed in order to ease the translation of users' intentions into subsequent actions. That is, that distal (I want to achieve X), proximal (so I want to do Y) and motor (so I'm doing Z) intentions interlock with the structure (the temporal and spatial constraints that allow a specific order and organization of action) and the interface of the telerehabilitation system (34, 35). The goal of dovetailing the system's features and functions with users' intentions can be achieved adopting a User-Centered Design (UCD) methodological approach, that is, involving users in the design of the telerehabilitation system from the very first stages of its development. The relevance of this methodological framework has been recognized in the literature for over two decades, and is considered the gold standard also in the design and development of healthcare technological solutions, including telerehabilitation systems (36, 37).

Focusing on different types of chronic disability, results showed that people with neuromotor disability (PD patients) experienced lower usability than COPD participants, which were predominantly characterized by physical disability. It has to be mentioned that PD presents neurological motor disability—including bradykinesia, resting tremor, and rigidity—since the early stages of the disease, plausibly affecting the interaction with technological devices. Moreover, beyond the well-known motor symptoms, individuals with PD frequently experience a wide range of non-motor symptoms from the prodromal phase, including cognitive impairment (38–41). The recent review of Schneider and Biglan highlights that both physical and cognitive limitations in PD could make interaction with healthcare technologies difficult (42). Interestingly, subjects included in the present study did not report relevant cognitive impairment. Nonetheless, technical requirements of a telerehabilitation system should be identified from the very beginning of its design and development, so as to make opportunities of action (i.e., the system's specific affordances) available even in the face of disabilities related to different clinical conditions. The goal of the UCD methodological framework is in fact to ensure that no aspect of the user experience takes place in the interaction with the technological system outside the designer's vision, this last being nurtured by the active and iterative involvement of users with different clinical profiles along the whole development process (43).

Moving to acceptability, results from the TAM questionnaire (15, 16) showed that all participants reported a high acceptance of the telerehabilitation system in terms of both perceived ease of use and perceived usefulness. In detail, subjects showed a high intention to use the system (“*Assuming I had access to SIDERA*^∧^*B, I intend to use it”*), evaluating it both easy to use (i.e., “*I find the tool to be easy to use*”) and useful and relevant for their health (“*the tool will improve my productivity*”). This result ties the patients' usability experience with their intention to use the system (behavioral intention) in light of the perceived utility for their own health condition, in accord with validated acceptability models (15).

Interestingly, considering separately the three different types of chronic disabilities, different patterns emerged. First of all, in all clinical conditions, behavioral intention is influenced by perceived usefulness. On the contrary, the perceived usability affects behavioral intention only in COPD patients. This means that the key factor influencing the use of a telerehabilitation system seems to be the belief that the technological solution could lead to advantages in performing the rehabilitation program better.

Following the most recent literature's evidence from Tsertsidis and colleagues (2019) on the influence of external factors (such as demographic characteristics, technological expertise, and disability level) on technology acceptance (17), we tested their role on the two main beliefs influencing the behavioral intention, the perceived usefulness and the perceived ease of use. Interestingly, in the whole sample, age and disability affect the perceived ease of use but not the perceived usefulness. Therefore, external factors such as age and the type of chronic disability do not affect the belief that the technological tool is capable of being used advantageously to enhance patients' health conditions. On the contrary, people with increased age and high disability levels (while giving usability scores above the cut-off) are likely to judge the telerehabilitation system as less usable than younger participants and people with lower levels of disability. Instead, the technological expertise and the educational level had no impact on the perceived ease of use, suggesting that the telerehabilitation system may be easily used also by people with medium-low levels of technological expertise or education.

Different trends appeared when considering the three clinical populations separately. Specifically, the perceived usability and usefulness are influenced by external factors differently depending on the type of chronic disability. In more detail, the perceived usability is affected by demographic characteristics. However, age is the only external factor influencing behavioral intention, only in COPD subjects, being solely tied with perceived usefulness. As regards technological expertise, the level of familiarity with technology has only an impact on perceived usefulness and therefore indirectly on the behavioral intention in the CHF group. Interestingly, the level of disability did not show a role on the main beliefs in any of the clinical conditions. Overall, these results suggest that the user experience toward digital solutions in chronic patients is only partially dependent on patient-specific characteristics. Rather, drawing on the UCD approach, the opportunity to dovetail user's intentions with rehabilitation systems' features and functions, highlights how the system's design involving users in the whole development process is paramount to reach a satisfactory user experience. This way, demographics and patient-specific characteristics are embedded in the optimization of the system's development process, rather than turning into potential barriers for its adoption (as when the system's evaluation is carried out mainly at the endpoint of design and development) (44).

When considering the acceptability model from Hirani and colleagues (2017) specifically focused on the acceptability of telehealth solutions, our results are aligned with the abovementioned acceptability results (18). Specifically, our participants perceived saving time, greater access and continuity of care, improved health and easier contact with professionals, and active involvement in their care management. Based on these results, the telerehabilitation system could be considered a valid substitute for face-to-face consultations, highly recommended to people with similar conditions.

Overall, our usability and acceptability results support the effectiveness (i.e., the possibility for the users to achieve goals), efficiency (i.e., users' efforts to reach the aim), and satisfaction (“*I think I would like to use this system frequently*”) of the telerehabilitation system. Specifically, in the present study, high levels of satisfaction were revealed by the SUTAQ. Several studies highlighted the key role of satisfaction in determining the success of telerehabilitation due to the ability of the system in reducing time and costs and giving positive health benefits to patients (45). Notably, a highly satisfying experience in rehabilitation is also linked to the patient's motivation and engagement in carrying out the prescribed activities (46). As suggested by the literature, the extensive library of digital content included in the telerehabilitation program may play a crucial role in supporting engagement and motivation during the course of the rehabilitation (5, 46). This is particularly important given that patients' engagement in telerehabilitation, especially in the asynchronous modality, is considered a primary aim to assure adherence to treatment and achieve clinical and functional outcomes.

Finally, our results support the role of external factors in determining the patient's acceptance or rejection of a digital health solution, as argued by the most recent models in the field. For this reason, researchers, clinicians and developers should design technological solutions according to those factors that, in addition to the perceived ease to use and the perception of usefulness, influence the system use, such as age, technological expertise and the type and level of disability. To this goal, as extensively claimed in the literature, stakeholders would highly benefit from the adoption of methodological frameworks (UCD approach) that enable the active involvement of patients from the very initial stages of a rehabilitation system's development. Drawing on this methodological option, the evaluation of patient's user experience does not refer, primarily, to a packed-up, full-fledged telerehabilitation system, but with the knowledge that designers can build up from patients' reiterated interactions with the system in its development process to embed such evaluations in an experience design able to attune targeted users' intentions to specific system's structure and features.

This study is not without limitations. Firstly, the influence of external factors on behavioral intention has been explored using correlations. Further studies should be conducted to deepen the role of external factors on Perceived Usefulness, Perceived Ease of Use, and Behavioral Intention using Structural Equation Modeling (SEM) in a larger sample. Moreover, a specific study should be conducted to validate the TAM model in chronic populations. Additionally, no verbal feedback on user experience has been collected from participants. Future studies should integrate the standardized questionnaires administered in the present study with qualitative interviews or think-aloud protocol methodologies to gain additional feedback on the patient's experience while using digital health technologies.

## Conclusion

The ongoing digital transformation requires clinicians and patients to ride the revolution of healthcare. Our user-experience results showed high usability and acceptability of the asynchronous telerehabilitation system SIDERA^∧^B. These findings support the efficiency and suitability of the telerehabilitation system in the modern digital transition of rehabilitation from inside to outside the clinic. Interestingly, our results suggest that the user experience toward this digital solution in chronic patients is partially correlated with patient-specific characteristics. These can be considered in the optimization of the entire development process of a digital solution, rather than turning into potential barriers to its adoption.

## Data availability statement

The raw data supporting the conclusions of this article will be made available upon reasonable request by the corresponding author.

## Ethics statement

The studies involving human participants were reviewed and approved by the IRCCS Fondazione Don Carlo Gnocchi-Milan Ethics Committee. The patients/participants provided their written informed consent to participate in this study.

## Author contributions

FBa, EF, and OR conceived the study. FR and FBo collected data and carried out the study. FBo and SI performed the statistical analysis. FR, FBo, and SI wrote the first draft of the manuscript. OR, EF, EG, and FBa substantively revised and edited the draft of the manuscript. All authors read and approved the final version of the manuscript.
